# An Investigation of Body Diode Reliability in Commercial 1.2 kV SiC Power MOSFETs with Planar and Trench Structures

**DOI:** 10.3390/mi15020177

**Published:** 2024-01-25

**Authors:** Jiashu Qian, Limeng Shi, Michael Jin, Monikuntala Bhattacharya, Atsushi Shimbori, Hengyu Yu, Shiva Houshmand, Marvin H. White, Anant K. Agarwal

**Affiliations:** 1 Department of Electrical & Computer Engineering, The Ohio State University, Columbus, OH 43210, USA; shi.1564@osu.edu (L.S.); jin.845@osu.edu (M.J.); bhattacharya.119@osu.edu (M.B.); yu.3868@osu.edu (H.Y.); houshmand.3@osu.edu (S.H.); white.1829@osu.edu (M.H.W.); agarwal.334@osu.edu (A.K.A.); 2 Ford Motor Co., Dearborn, MI 48124, USA; ashimbor@ford.com

**Keywords:** SiC, MOSFET, body diode, reliability, basal plane dislocation, planar, trench, P+ implantation, deep JFET

## Abstract

The body diode degradation in SiC power MOSFETs has been demonstrated to be caused by basal plane dislocation (BPD)-induced stacking faults (SFs) in the drift region. To enhance the reliability of the body diode, many process and structural improvements have been proposed to eliminate BPDs in the drift region, ensuring that commercial SiC wafers for 1.2 kV devices are of high quality. Thus, investigating the body diode reliability in commercial planar and trench SiC power MOSFETs made from SiC wafers with similar quality has attracted attention in the industry. In this work, current stress is applied on the body diodes of 1.2 kV commercial planar and trench SiC power MOSFETs under the off-state. The results show that the body diodes of planar and trench devices with a shallow P+ depth are highly reliable, while those of the trench devices with the deep P+ implantation exhibit significant degradation. In conclusion, the body diode degradation in trench devices is mainly influenced by P+ implantation-induced BPDs. Therefore, a trade-off design by controlling the implantation depth/dose and maximizing the device performance is crucial. Moreover, the deep JFET design is confirmed to further improve the body diode reliability in planar devices.

## 1. Introduction

Compared to silicon, silicon carbide (SiC) power MOSFETs are favored in the power electronics market, especially in electric vehicles (EVs), due to their lower switching loss, higher temperature capability, and higher switching frequency [1]. The initial and still most widely used commercial SiC power MOSFETs are conventional planar MOSFETs [2]. However, the SiC/SiO_2_ interface is more complex than the Si/SiO_2_ interface, causing the gate oxide to have a higher density of interface states and near-interface traps during thermal growth [3,4]. Additionally, a high density of crystallographic and surface defects in the SiC substrate and the epitaxial layer during high-temperature growth negatively affects the reliability of SiC power MOSFETs [5,6]. Thus, the commercialization of SiC power MOSFETs presents significant challenges. As the thermal growth technology of the gate oxide layer and the epitaxial layer has gradually matured, the reliability of planar SiC power MOSFETs has significantly improved [7,8,9,10,11,12]. The degradation of the body diode has become negligible for voltage ratings below 1.2 kV, and some vendors have even eliminated the impact of body diode degradation at the voltage rating of 3.3 kV [13,14]. This allows the parasitic body diode to be directly used as a free-wheeling diode, replacing the external Schottky diode and thus significantly reducing the production cost [15]. With the relative simplicity and maturity of the manufacturing process for planar SiC power MOSFETs, more electric vehicle OEMs and tier-one suppliers are adopting them in their onboard chargers and drivetrain inverters [16].

Currently, in the electric vehicle market, battery voltages are mainly around 400 V, causing 650 V SiC power MOSFETs to occupy a large market share [17,18]. However, for a higher driving range, faster charging efficiency, and better performance, more and more electric vehicle OEMs are considering upgrading from 400 V systems to 800–900 V systems, shifting the market’s focus to 1.2 kV products [19,20,21]. Due to reliability and cost, design trends for power modules favor reducing the number of discrete devices while maintaining or even increasing the current rating [22]. This sets higher demands on the on-resistance ($R_{DS\_ON}$) of SiC power MOSFETs. Furthermore, with the rapid expansion of electric vehicles in the automotive market, the demand for SiC power MOSFETs production capacity is increasing; meanwhile, the performance and structural limitations of planar SiC power MOSFETs are becoming apparent [23]. Therefore, trench SiC power MOSFETs, with their higher channel electron mobility, elimination of the JFET resistance, and smaller cell pitch, are gaining attention in the market [24,25,26]. These features make achieving higher current ratings and production capacity for trench SiC power MOSFETs possible.

However, unlike conventional planar SiC power MOSFETs, under the blocking state, trench SiC power MOSFETs experience a high electric-field crowding at the bottom corners of the trench gate oxide, subjecting the gate oxide to higher stress [26,27]. This may require the derating of the drift region, which offsets the $R_{DS\_ON}$ gains of the trench structure, and even lead to premature breakdown, affecting the reliability of trench SiC power MOSFETs [25,28,29]. To protect the trench gate oxide, P-type implants for shielding the gate trench corners have become a consensus in the design of trench SiC power MOSFETs [30]. However, for adequate protection, P-type implants in trench SiC power MOSFETs usually require deeper implantation and higher doses than the P-body in planar SiC power MOSFETs [28]. This may sacrifice the reliability of the parasitic body diode, necessitating a reassessment of whether to use an external Schottky diode as a free-wheeling diode. Trench SiC power MOSFETs, due to their relatively complex process, may not have the cost advantage of planar SiC power MOSFETs [31]. Integrating an external Schottky diode may exacerbate this cost disadvantage, eroding the benefits provided by the smaller cell pitch of trench SiC power MOSFETs [32]. Furthermore, there are variations in the characteristics of P-type implants among different vendors and even different generations of trench SiC power MOSFETs from the same vendor, making the research on the reliability of the body diode in SiC power MOSFETs essential. This work investigates and compares the body diode reliability of 1.2 kV commercial products from the mainstream vendors of SiC power MOSFETs with both planar and trench structures and proposes methods to enhance the reliability of the parasitic body diode.

## 2. Materials and Methods

### 2.1. Devices under Test (DUTs)

The devices under test (DUTs) used in this work are all 3-pin TO-247 packaged commercial 1.2 kV SiC power MOSFETs, without integrated Schottky diodes, as shown in Table 1. Among them, devices from Vendors E, I, and C are all planar SiC power MOSFETs, while those from Vendors K and D are trench SiC power MOSFETs. The planar structures of the devices from Vendors E, I, and C show the same structure. However, the trench structures of the devices from Vendors K and D differ, reflected in the number and depth of trenches within the cell pitch. Vendor K uses a single-trench structure (gate trench only), whereas Vendor D utilizes a double-trench structure (gate trench and source dummy trench), with slight differences between the third and fourth generations.

**Figure 1:**
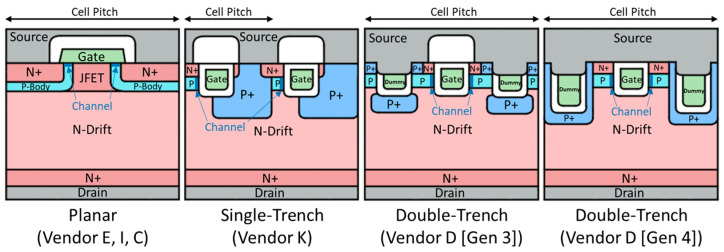
The cross-sectional schematics of the planar structure, single-trench structure, and double-trench structure of two generations from left to right.

Figure 1 depicts the conventional planar structure of the devices from Vendors E, I, and C, and the trench structures of the devices from Vendors K, D (Gen 3), and D (Gen 4). It can be observed that the single-trench structure of Vendor K’s devices boasts a significant advantage in the cell pitch. However, each single gate trench has a channel on only one side, with the other side covered by a deep P+ implant to protect the trench corners. This means that every channel in devices from Vendor K has to work harder. For devices from Vendor D (Gen 3) with their gate trench plus source trench to form the double-trench configuration, as shown in the cross-sectional SEM images of their bare dies, their structure may have no obvious advantages of the cell pitch over the planar structure, because each cell has its own source trenches which are not shared with adjacent cells [28]. Differing from Vendor K’s deep P+ implants, the P+ implantation in devices from Vendor D (Gen 3) is carried out within the trenches after their formation. This suggests a shallower P+ depth in devices from Vendor D (Gen 3) compared to those from Vendor K, possibly sacrificing some gate oxide protection. However, having source trenches and corresponding P+ implants on both sides of each gate trench ensures channels on both sides, alleviating the channel stress. Therefore, in the structure of the devices from Vendor D (Gen 4), the double trench is retained. Also, the cross-sectional SEM images of their bare dies indicate that adjacent cells begin to share a source trench, improving the cell pitch [28]. Simultaneously, the P+ implants are modified to match Vendor K’s deep P+ implants, and the sequence of source trench etching and P+ implantation is swapped. The etching depth of the source trench wrapped in deep P+ in Gen 4 devices is doubled, offering better protection to the gate oxide than Gen 3 devices.

### 2.2. Experimental Methods

In this work, the reliability of the body diodes of DUTs was evaluated through the long-term constant current stress test. The sample size for each vendor’s devices was ten. These ten DUTs were connected in series and subjected to a constant current stress that matched their current rating. Meanwhile, a −5 V bias was uniformly applied to the gate of all the DUTs, as this is the recommended operational voltage for the body diode on device datasheets. To determine whether the body diode had a degradation trend that exceeded the industrial degradation criterion (to be introduced in Section 3) and determine the test efficiency, the body diode stress time for all the DUTs was set to 100 h. Electrical measurements were conducted before the stress, 20 h after, 50 h after, and 100 h after, respectively, to observe the changes in body diode characteristics and their impact on device performance. The electrical measurements included the third-quadrant $I_{D}-V_{D}$ body diode characteristics (with a gate bias of −5 V) to extract the body diode’s forward voltage drop ($V_{F}$) @ $I_{DS}$ = −20 A; the first-quadrant $I_{D}-V_{D}$ output characteristics (with a gate bias of 20 V) to determine $R_{DS\_ON}$ at $V_{DS}$ = 0.5 V; and the first-quadrant $I_{D}-V_{D}$ forward blocking characteristics (with a gate bias of 0 V) to observe the forward leakage current and drain-to-source breakdown voltage. The degradation of the body diode manifested in the change rate of $V_{F}$ ($\delta V_{F}/V_{F0}$, the percentage change in $V_{F}$ compared to its pre-stress value), the change rate of $R_{DS\_ON}$ ($\delta R_{DS\_ON}/R_{DS\_ON0}$, the percentage change in $R_{DS\_ON}$ compared to its pre-stress value), and changes in the blocking characteristic curves. Throughout the constant current stress process, the ten DUTs connected in series were mounted on a cold plate. An electrically insulating thermal pad separated the metal plate in the TO-247 package (connected to the drain) of all the DUTs from the metal cold plate. The cold plate was then connected to an industrial-grade water chiller to effectively dissipate the heat generated inside the device package during the stress, thereby stabilizing the junction temperature.

## 3. Results

In this section, the results of the 100 h body diode stress tests conducted on the selected commercial SiC MOSFETs are presented. Ideally, SiC power MOSFETs would exhibit no rate of change (ROC) in their body diode characteristics after undergoing stress. However, in reality, devices display varying degrees of ROCs after the same stress. To differentiate between devices whose ROCs remain within and outside the acceptable limits for their application scenarios after stress, a widely used industrial degradation criterion is necessary. Currently, there is no official criterion, but a commonly used one that involves changes in $V_{F}$ or $R_{DS\_ON}$ exceeding about +5% from their initial values has been extensively employed in previous research on body diode degradation [33,34,35]. In this work, we continued to use this industrial degradation criterion for analysis. All ROCs over different time points were based on the average levels of ROCs of all the surviving devices at these time points. Due to the randomness of measurement errors, some ROCs appeared as negative values but were within acceptable limits. For easy observation, changes in the blocking characteristic curves were compared only between the start of the stress and the end of the 100 h stress, based on the most representative device among those that survived until the end. The dotted lines represent the blocking characteristic curves before the stress, and the solid lines represent them after 100 h of stress.

### 3.1. Body Diode Stress Test Results of Commercial Planar SiC Power MOSFETs

**Figure 2:**
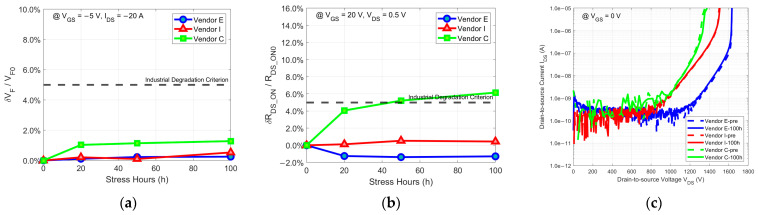
The body diode stress test results of (**a**) $\delta V_{F}/V_{F0}$, (**b**) $\delta R_{DS\_ON}/R_{DS\_ON0}$, and (**c**) the changes in blocking characteristic curves for commercial planar SiC power MOSFETs from Vendors E, I, and C.

Figure 2 displays the 100 h body diode stress test results for commercial planar SiC power MOSFETs from the three selected vendors. Compared to the industrial criterion for body diode degradation, it can be observed that, regardless of the ROC in $V_{F}$ or ROC in $R_{DS\_ON}$ of the body diode, the DUTs from two of the three vendors remain far away from the industrial degradation criterion and very close to the ideal conditions throughout the stress. Only the DUTs from Vendor C slightly exceed the industrial degradation criterion. Moreover, their blocking characteristic curves demonstrate high stability, with no significant changes in the forward leakage current or drain-to-source breakdown voltage before and after the stress. As a result, body diode degradation is no longer a significant issue for 1.2 kV planar SiC power MOSFETs, suggesting that the parasitic body diode of planar SiC power MOSFETs at this voltage rating can be considered for application as a free-wheeling diode, reducing the production cost and increasing the production capacity. Regarding the DUTs from Vendor C with body diode degradation slightly exceeding the industrial degradation criterion compared to the DUTs from the other two vendors, the possible reasons require further analysis of the stacking faults (SFs) that lead to degradation. By examining the location and propagation trends of SFs, it is possible to determine the initial positions of basal plane dislocations (BPDs) and thus analyze the sources of BPDs. The sources of BPDs will be introduced in Section 4. According to the sources of BPDs, the causes of degradation can be deduced in the corresponding manufacturing process. Thus, if Vendor C wants to further enhance the reliability of their body diodes to match the leading products on the market, they may need to make improvements in the device structure or process, which will be discussed in Section 4.3.1.

### 3.2. Body Diode Stress Test Results of Commercial Trench SiC Power MOSFETs

**Figure 3:**
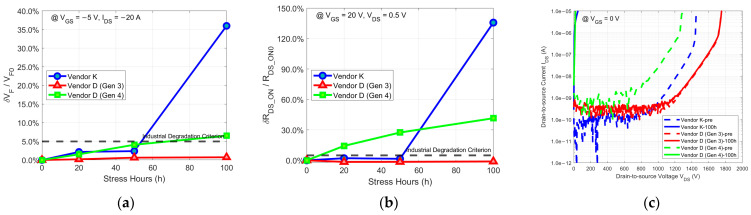
The body diode stress test results of (**a**) $\delta V_{F}/V_{F0}$, (**b**) $\delta R_{DS\_ON}/R_{DS\_ON0}$, and (**c**) the changes in blocking characteristic curves for three types of commercial trench SiC power MOSFETs from Vendors K and D.

Figure 3 shows the 100 h body diode stress test results for three types of commercial trench SiC power MOSFETs from the two selected vendors. Unlike the commercial planar SiC power MOSFETs, when comparing their characteristics against the industrial criterion for body diode degradation, the devices from both Vendor K and Vendor D (Gen 4) exhibit ROCs in $V_{F}$ and $R_{DS\_ON}$ of the body diode that exceed the industrial degradation criterion. The ROCs for devices from Vendor K exceed the industrial degradation criterion after 20 h of stress and exhibit more severe degradation after 50 h. Only the devices from Vendor D (Gen 3) maintain ROCs far below the industrial degradation criterion throughout the stress, demonstrating reliability similar to that of advanced commercial planar SiC power MOSFETs. Concurrently, changes in their blocking characteristic curves also mirror the level of their body diode degradation. The devices from Vendor K and Vendor D (Gen 4) exhibit very “leaky” characteristics in their blocking characteristic curves after 100 h of stress, with the forward leakage current reaching the device breakdown level at a very low drain-to-source voltage. Only the devices from Vendor D (Gen 3), due to the high reliability of their body diodes, display the same high stability in their blocking characteristics as the commercial planar SiC power MOSFETs. Therefore, body diode degradation remains a challenge for 1.2 kV trench SiC power MOSFETs. Although some trench structures, like those of the devices from Vendor D (Gen 3), offer relatively stable body diodes that could be directly used as free-wheeling diodes, this may be achieved at the expense of sacrificing the device performance to a certain extent. Hence, for most trench SiC power MOSFETs with high performance, integrating external Schottky diodes as free-wheeling diodes may still be needed, which negatively impacts the production cost and capacity.

**Figure 4:**
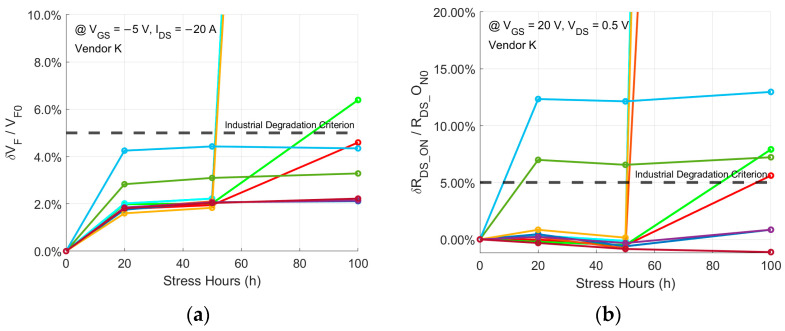

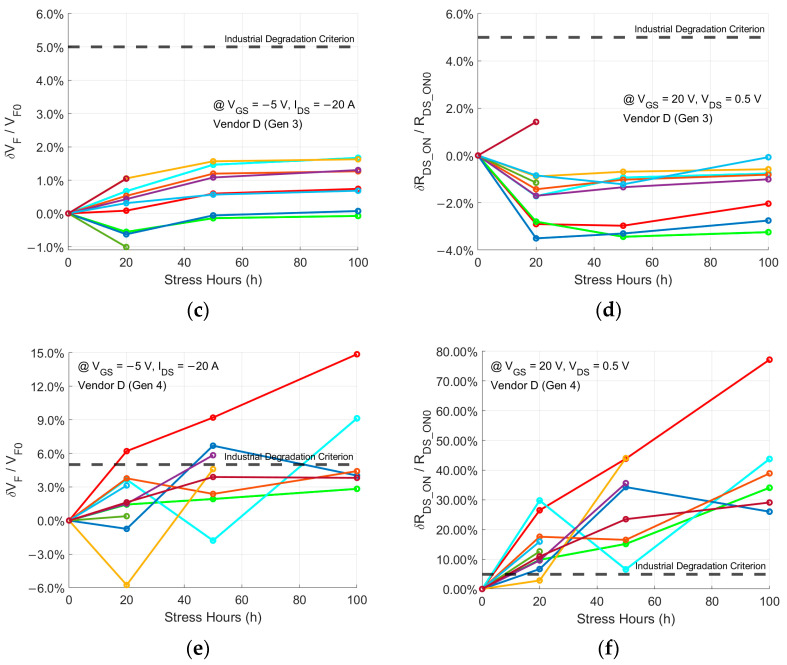
The 100 h body diode stress test results of (**a**) $\delta V_{F}/V_{F0}$ and (**b**) $\delta R_{DS\_ON}/R_{DS\_ON0}$ for ten trench DUTs from Vendor K; (**c**) $\delta V_{F}/V_{F0}$ and (**d**) $\delta R_{DS\_ON}/R_{DS\_ON0}$ for ten trench DUTs from Vendor D (Gen 3); (**e**) $\delta V_{F}/V_{F0}$ and (**f**) $\delta R_{DS\_ON}/R_{DS\_ON0}$ for ten trench DUTs from Vendor D (Gen 4). Each color line represents one discrete DUT from ten.

Figure 4 depicts the 100 h body diode stress test results for all the DUTs of the three types of commercial trench SiC power MOSFETs. As shown in Figure 4a,b, all ten DUTs from Vendor K survive the 100 h stress, with seven exceeding the industrial degradation criterion after the stress and three exhibiting very severe degradation. Figure 4c,d indicate that two out of the ten DUTs from Vendor D (Gen 3) fail during the 100 h stress, yet all the DUTs remain below the industrial degradation criterion throughout the stress. Figure 4e,f reveal that four out of the ten DUTs from Vendor D (Gen 4) fail during the stress, with all the DUTs exceeding the industrial degradation criterion after the stress. The yields of qualified body diodes after the stress for all the vendors of the aforementioned commercial trench SiC power MOSFETs are summarized in Table 2. The bad yields further prove that integrating external Schottky diodes as free-wheeling diodes is still necessary for most trench SiC power MOSFETs. For comparison, the body diodes of the devices from all the vendors of commercial planar SiC power MOSFETs survive the 100 h stress and remain below the industrial degradation criterion during the stress.

## 4. Discussion

This section involves the analysis of the body diode stress test results of commercial SiC power MOSFETs with different structures. The results confirm that the mechanism causing the body diode degradation in SiC MOSFETs is the expansion of BPD-induced SFs by absorbing the activation energy from the recombination of injected holes and electrons in the drift region [36]. Hence, the key to analyzing the body diode stress test results in this work lies in the source of the BPDs. The primary sources of BPDs include (1) BPDs originating from the substrate that propagate into the drift region; (2) BPDs formed in the epitaxial layer (i.e., the drift region) during epitaxial growth; (3) BPD clusters formed due to inclusions (SiC down-fall); (4) small and tight BPD clusters formed around micropipes; (5) BPDs formed near the implanted region after P-type implantation and activation annealing [34]. A series of process improvements have been made, such as introducing a recombination-enhanced buffer layer between the substrate and the drift region, a KOH etching process before the conventional epitaxial growth to create BPD etch pits for enhancing the conversion of BPDs to threading edge dislocations (TEDs), and using ultraviolet photoluminescence (UVPL) on wafers to screen out dies with BPDs [37,38,39,40]. Stahlbush et al. have demonstrated the high quality of commercial SiC wafers with an epi-layer thickness of 10 µm suitable for the 1.2 kV rating, and some vendors have achieved a near-equivalent quality of SiC wafers for the 3.3 kV rating with a 30 µm epi-layer thickness [34]. Thus, the body diode degradation phenomenon observed in this work is believed to be caused by the BPDs formed near the implanted region after P-type implantation and activation annealing [38].

### 4.1. Analysis of Body Diode Stress Test Results of Commercial Planar SiC Power MOSFETs

The devices from Vendors E, I, and C share the same planar structure, as shown in Figure 1. In this planar structure, P-type implantation is of low dose and energy, used to form the shallow P-body. Stahlbush et al. have shown that the low-dose P-type implantation in SiC, even after activation annealing, does not create significant BPDs near the implanted region [41]. Thus, the commercial 1.2 kV planar SiC power MOSFETs tested in this work, based on the inherently low BPD density in the SiC epi-layer, do not introduce new implantation-induced BPDs with the low-dose and low-energy P-type implantation. As a result, they exhibit high reliability and almost no degradation during the long-term constant current stress. Considering that devices from Vendor C exhibit more pronounced degradation, slightly exceeding the industrial degradation criterion compared to those from Vendors E and I, there is one possible reason that the P-type implantation for devices from Vendor C is conducted at room temperature. This could be a strategy to reduce the production cost, but as a trade-off, P-type implantation at room temperature may have a higher probability of introducing new BPDs post-activation annealing than heated implantation. Qian et al. have previously shown that SiC power MOSFETs with P-type implantation at 500 °C, compared to those with P-type implantation at room temperature, exhibit almost negligible body diode degradation [14]. The difference in body diode degradation at these two temperatures aligns with the observed difference in this work between devices from Vendor C and those from Vendors E and I, leading to the belief that this variation is possibly due to different implantation temperatures and/or different annealing conditions.

### 4.2. Analysis of Body Diode Stress Test Results for Commercial Trench SiC Power MOSFETs

Devices from Vendor K, Vendor D (Gen 3), and Vendor D (Gen 4) possess distinct trench structures, as depicted in Figure 1. Compared to devices from Vendor K and Vendor D (Gen 4), despite undergoing a high-dose P+ implantation process, the P+ implants in Vendor D (Gen 3) devices are shallower, thus requiring less implantation energy. This significantly reduces the likelihood of BPD formation after activation annealing. When controlling the implantation depth and performing high-temperature implantation, the reliability of the parasitic body diode can reach the levels observed in advanced commercial planar SiC power MOSFETs. However, the shielding function that the P+ implants provide to the gate trench corners also becomes diminished. This necessitates the derating of the drift region by increasing its thickness, resulting in an increase in $R_{DS\_ON}$. Figure 3c confirms this, as the drain-to-source breakdown voltage of the devices from Vendor D (Gen 3), nearing 1.8 kV, surpasses that of Vendor K and Vendor D (Gen 4). By contrast, devices from both Vendor K and Vendor D (Gen 4) may have introduced a large number of implantation-induced BPDs, due to the high-dose and high-energy deep P+ implantation for fully protecting the gate trench corners, thereby significantly sacrificing the reliability of the parasitic body diode. Stahbush et al. suggest that, although the formation of BPDs would be enhanced at the corners of the reactive ion etching (RIE) region, oxidation eliminates surface damage at the corners and suppresses the formation of BPDs [42]. Therefore, the trench structure formed by RIE is influenced by oxidation, which inhibits the formation of RIE-induced BPDs, meaning that the main source of BPDs causing the body diode degradation in this work is still P-type implantation. Devices from Vendor D (Gen 3) with the RIE-formed trench structure, yet still possessing highly reliable body diodes, provide strong proof for this. It is worth noting that since the deep P+ implants of the devices from Vendor K occupy a larger proportion in their cell pitch compared to those of the devices from Vendor D (Gen 4), the density of implantation-induced BPDs in devices from Vendor K may be higher than that in devices from Vendor D (Gen 4). As a result, under constant current stress and upon absorbing sufficient activation energy, devices from Vendor K exhibit more severe body diode degradation than those from Vendor D (Gen 4). Also, SFs causing severe body diode degradation in both cases become generation centers when these devices are under the blocking state, leading to a significant increase in the forward leakage current, resulting in a very ‘leaky’ state.

### 4.3. Structural Improvements to Enhance the Body Diode Reliability

#### 4.3.1. Structural Improvements for Commercial Planar SiC Power MOSFETs

**Figure 5:**
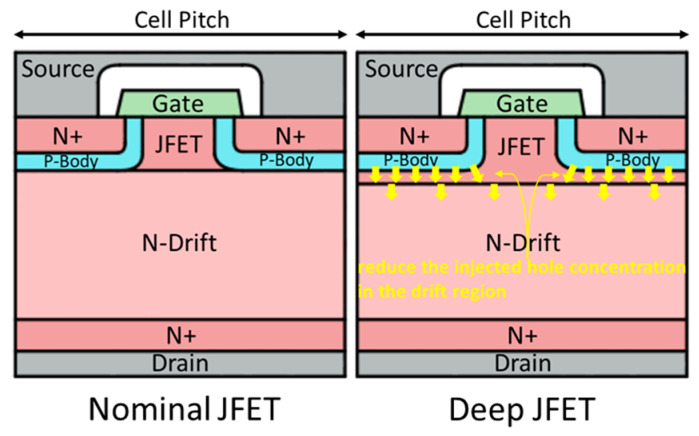
The cross-sectional schematics of the planar structure with a nominal JFET and deep JFET from left to right.

**Figure 6:**
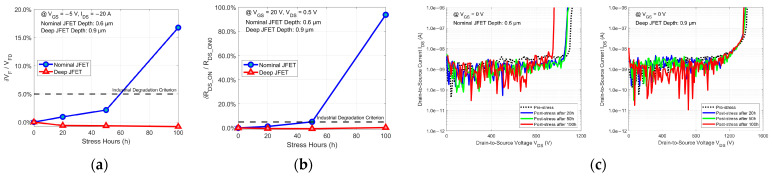
The 100 h body diode stress test results of (**a**) $\delta V_{F}/V_{F0}$, (**b**) $\delta R_{DS\_ON}/R_{DS\_ON0}$, and (**c**) the changes in blocking characteristic curves for the fabricated planar SiC power MOSFETs with the nominal and deep JFET.

Experimental results in this work indicate that most commercial planar SiC power MOSFETs exhibit excellent reliability in their body diodes. These high-reliability body diodes meet the requirements of some specific application scenarios as free-wheeling diodes. However, some vendors’ devices exhibit body diode degradation still slightly beyond the industrial degradation criterion after the prolonged constant current stress, and therefore they may not be as competitive in harsher environments compared to devices from other vendors with superior body diodes. Considering the high cost of heated ion implantation systems and the limited capacity of fabs running them, some structural improvements can also help enhance the body diode reliability. Previous analysis revealed that the SFs causing body diode degradation expand due to the absorption of the activation energy from the recombination of injected holes and electrons in the drift region, suggesting a strategy to suppress the degradation by reducing this recombination in the drift region. Figure 5 shows a design with a deep N-type-doped JFET in the conventional planar structure to reduce the injected hole concentration in the drift region, thus reducing the recombination in the drift region. Five 1.2 kV planar SiC power MOSFETs based on this deep JFET design were fabricated, and as a control group, another five with a nominal JFET were also fabricated. The general information on these fabricated DUTs is summarized in Table 3. Knowing the die dimensions of the fabricated devices for a die area of ~0.045 cm^2^, both sets of devices underwent stress at the same current density of ~467 A/cm^2^ for 100 h. The results of this 100 h stress test are shown in Figure 6. As illustrated in Figure 6a,b, even though they are planar SiC power MOSFETs, the body diode degradation of these lab-grade devices with a nominal JFET still exceeds the industrial degradation criterion. In contrast, degradation in devices with a deep JFET is significantly suppressed, achieving reliability similar to the body diodes of advanced commercial planar SiC power MOSFETs. Moreover, the variations in the representative blocking characteristics in Figure 6c also demonstrate that the deep JFET structure suppresses body diode degradation, achieving very stable blocking characteristics, unlike the noticeable degradation observed in devices with the nominal JFET structure. Thus, the deep JFET design can be considered an effective structural improvement for enhancing the body diode’s reliability.

#### 4.3.2. Structural Improvements for Commercial Trench SiC Power MOSFETs

For commercial trench SiC power MOSFETs, due to their unique structure, the deep P+ implantation is crucial for adequately protecting the gate trench corners. However, the excessive P+ implantation depths and doses often come at the expense of body diode reliability. Devices from Vendor D (Gen 3) offer insights into maintaining the body diode reliability in a trench structure. Since mainly the bottom corners of the gate trench require deep P+ protection, the direct deep P+ implantation from the device surface can be replaced by first performing RIE to approach the gate trench corners at both sides, followed by a shallower P+ implantation process. This approach ensures protection for the gate trench corners to a certain extent while minimizing the energy of the P+ implantation, reducing the introduction of implantation-induced BPDs. However, compared to the deep P+ implantation, there may be slightly sacrificed protection for the gate trench corners. This could necessitate the derating of the drift region, eroding the benefits of the trench design. Therefore, the structural improvements in commercial trench SiC power MOSFETs will be a trade-off. While ensuring that the dose and energy of the P+ implantation cause body diode degradation below the industrial degradation criterion, the depth of the pre-implantation RIE should be adjusted to ensure that the position of P+ can provide maximum protection for the gate trench corners, thus minimizing the derating of the drift region and maximizing the device performance.

## 5. Conclusions

This work explores the reliability of the parasitic body diodes in 1.2 kV commercial SiC power MOSFETs with both planar and trench structures. Through a sufficient sample size, the degradation of the body diode for each DUT was studied with the yields of qualified body diodes after the stress, for investigating the possibility of using the body diode as a free-wheeling diode. Due to the structural advantage and mature process, the parasitic body diode of 1.2 kV commercial planar SiC power MOSFETs presents high reliability, demonstrating or having realized the potential of direct application as a free-wheeling diode. Furthermore, structural improvements, such as a deep JFET design, can further enhance the reliability of the body diode to meet more stringent application scenarios. This helps reduce the production cost and improve the production capacity of 1.2 kV commercial planar SiC power MOSFETs. However, for 1.2 kV commercial trench SiC power MOSFETs, the high electric field crowding at the gate trench corners under the blocking state suggests that the shielding role of the deep P+ implants is required, adversely affecting the reliability of the body diode due to implantation-induced BPDs. Therefore, 1.2 kV commercial trench SiC power MOSFETs may still need to be integrated with external Schottky diodes as stable free-wheeling diodes for switching applications, negatively impacting their production cost and capacity. Some vendors of 1.2 kV commercial trench SiC power MOSFETs have achieved highly reliable body diodes by sacrificing some of the shielding function from the P+ implants on the gate trench corners, thereby eroding some of the device performance gains achieved by the trench structure. However, the trade-off design based on the P+ implantation depth, dose, and position will continue to be the research focus for 1.2 kV commercial trench SiC power MOSFETs aiming to use parasitic body diodes as free-wheeling diodes.

## Figures and Tables

**Table 1:** General information on the commercial DUTs in this work.

Vendor	Voltage Ratings (V)	Current Ratings (A)	Structure
E	1200	11	Planar
I	1200	10	Planar
C	1200	12	Planar
K	1200	13	Single Trench
D (Gen 3)	1200	17	Double Trench
D (Gen 4)	1200	26	Double Trench

**Table 2:** The yields of qualified body diodes after the stress for all the vendors of commercial trench SiC power MOSFETs.

Vendors of Devices	Yields of Qualified Body Diodes
K	30%
D (Gen 3)	100%
D (Gen 4)	0%

**Table 3:** General information on the fabricated DUTs with nominal and deep JFET in this work.

JFET Type	JFET Depth (µm)	Voltage Ratings (V)	Current Ratings (A)	Structure
Nominal	0.6	1200	21	Planar
Deep	0.9	1200	21	Planar

## Data Availability

Data are contained within the article.
